# Supplementary material for: Super-enhancer-guided mapping of regulatory networks controlling mouse trophoblast stem cells
Source: Nat Commun. 2019 Oct 18;10:4749. doi: 10.1038/s41467-019-12720-6 (PMC6802173; doi:10.1038/s41467-019-12720-6)
Supplement: Supplementary file 2 — Supplementary Information [file 41467_2019_12720_MOESM2_ESM.pdf]

Supplementary Information

Super-enhancer-guided mapping of regulatory networks controlling trophoblast stem cells

Lee et al.

Supplementary Figures

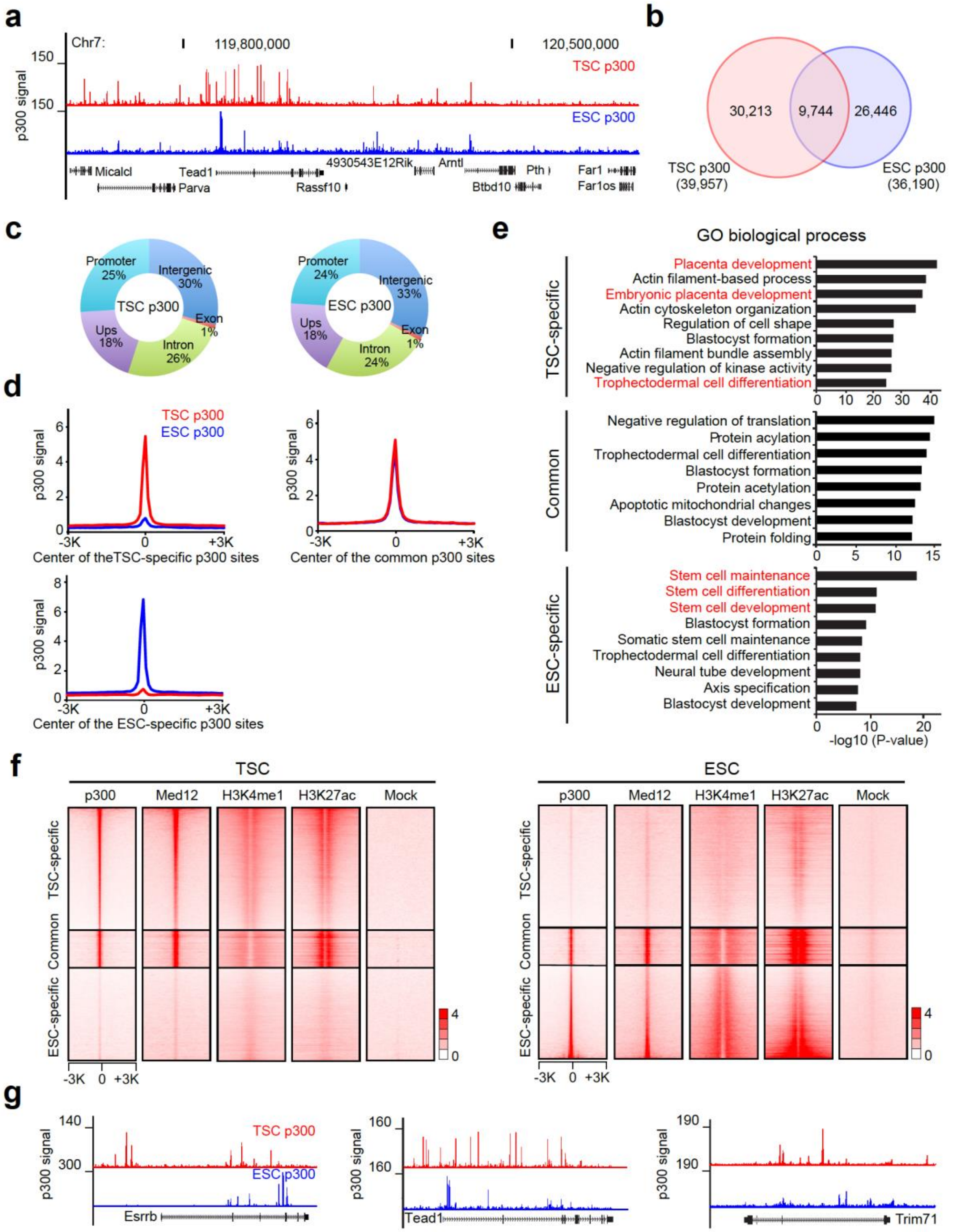

Supplementary Fig. 1. Identification of TSC-specific enhancers using p300 ChIP-seq. a. Signal track image showing occupancy of p300 in TSCs (red) and ESCs (blue). **b**, Venn diagrams presenting overlap of p300 binding sites between TSCs and ESCs. **c**, Pie charts presenting the distribution of p300 binding sites in ESCs (left panel) and TSCs (right panel). Promoters are

regions ±2 Kb from transcription start sites (TSSs); Upstream sites fall within regions between 2 Kb and 20 Kb upstream of TSSs; Intergenic sites fall within regions that are not classified as promoters, upstream, exons and introns. **d**, The distribution of p300 occupancy signals in TSCs and ESCs near the center of TSC-specific, common, and ESC-specific p300 binding sites. **e**, Gene ontology (GO) analysis of TSC-specific, common, and ESC-specific p300 sites. **f**, Heatmaps showing occupancy signals of p300, Med12, and mock control as well as signatures of H3K4me1 and H3K27ac in TSCs (left panel) and ESCs (right panel) near the center of all p300 peaks from both TSCs and ESCs. Overlap analysis of p300 binding sites separated three distinct classes of p300 sites (TSC-specific, common and ESC-specific) between TSCs and ESCs. **g**, Signal track images of p300 ChIP-seq in TSCs and ESCs.

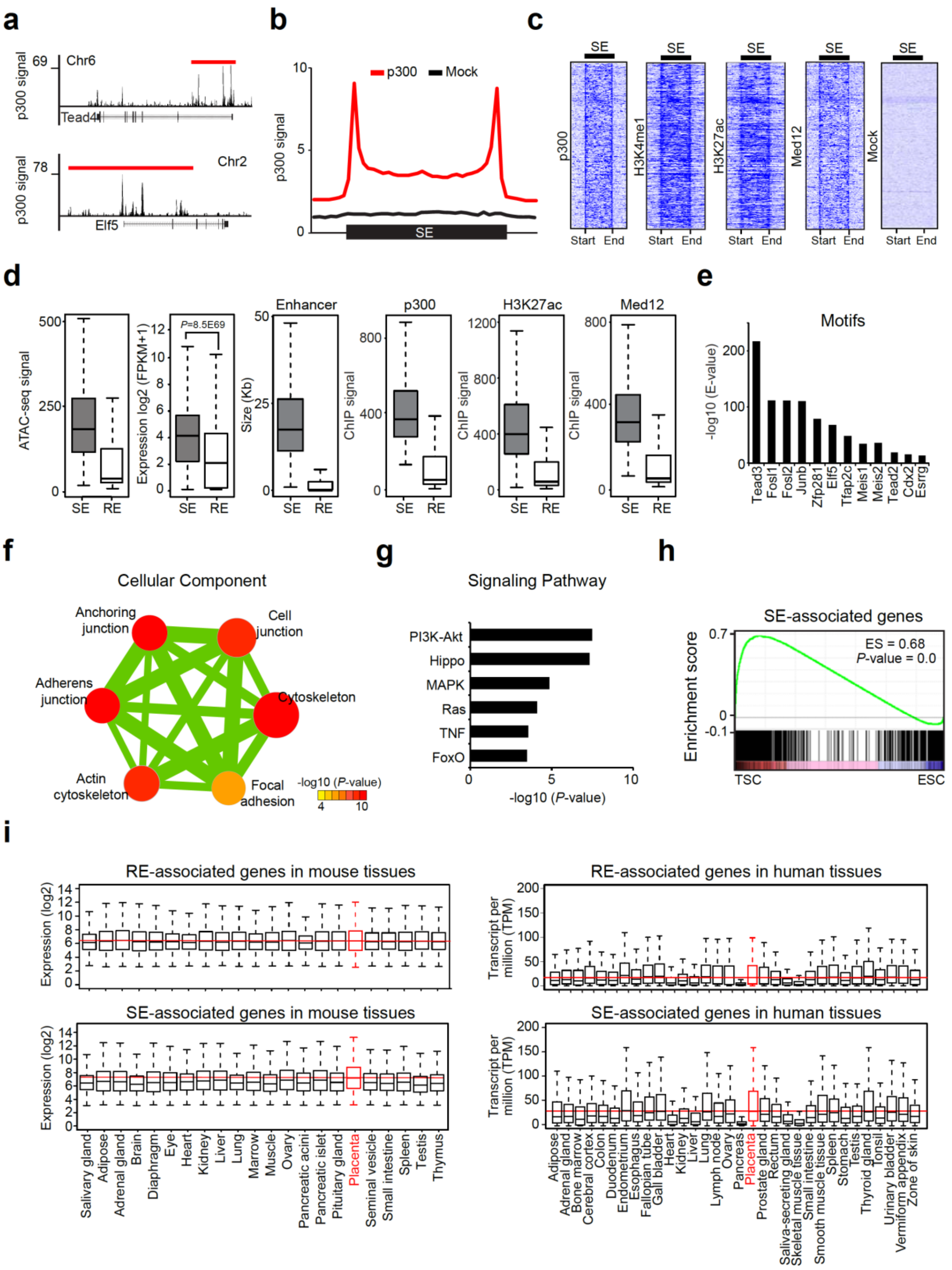

Supplementary Fig. 2. Characteristics of TSC-specific super-enhancers (SEs) and SE-associated genes. a, Signal track images of p300 depicting multiple enhancers associated with TSC-specific genes. Red bar indicates area of SEs. **b**, ChIP-seq signal of p300 around SEs. **c**,

Heatmaps showing p300, H3K4me1, H3K27ac, and Med12 signals of ChIP-seq in SEs. **d**, Boxplots showing chromatin accessibility, gene expression level, the size of enhancers, and ChIP-seq signals of p300, H3K27ac, and Med12 in SEs and regular enhancers (REs). **e**, A bar graph presenting enriched motifs within SEs. **f**, A network map illustrating enriched gene ontology (GO) terms of cellular component in SE-associated genes. Node color and line thickness indicate *P*-value and the extent of overlapped genes between enriched terms, respectively. **g**, A bar graph showing enriched signaling pathways for the SE-associated genes. **h**, Gene set enrichment analysis (GSEA) of SE-associated genes. NES indicates normalized enrichment value. **i**, boxplots showing the distribution of gene expression levels of mouse (left panel) and human orthologs (right panel), corresponding to RE-associated (upper panel) or SE-associated (lower panel) mouse genes, in various tissues.

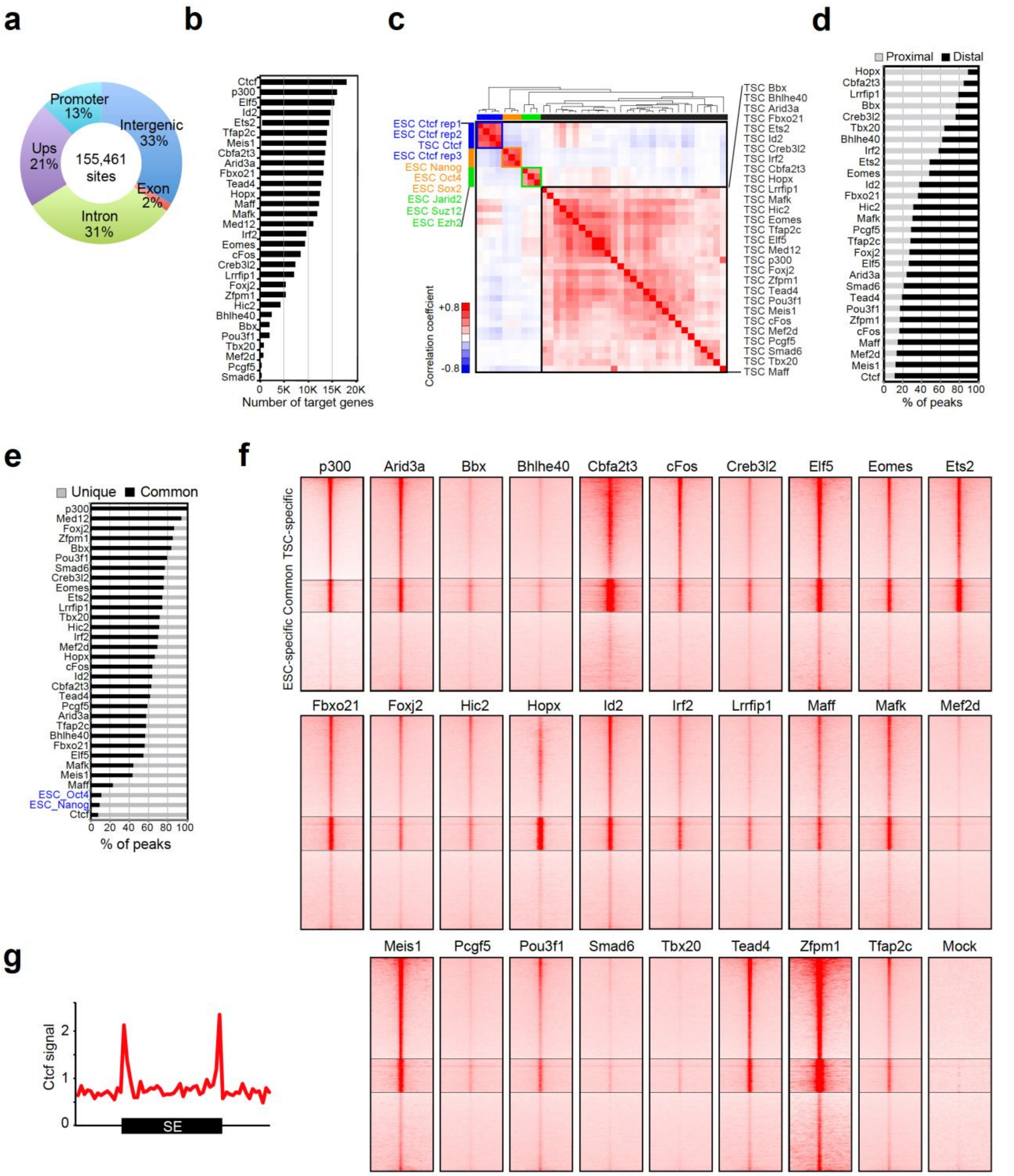

Supplementary Fig. 3. Occupancy patterns of TSC-specific-SE-associated TFs. a, A pie chart showing the distribution of cis-regulatory elements, found by ChIP-seq of TSC-specific TFs in TSCs, across the mouse genome. **b**, Total numbers of target genes corresponding to each TF. **c**, A correlation heatmap of various TFs' occupancy. Different functional classes of genes are color-coded (CTCF: blue, pluripotency factors: orange, PRC complex: green, and TSC-specific TFs:

black). Gradient color scale bar on the left indicates correlation coefficients. **d**, Percentage of proximal and distal sites in each TF's binding sites. Proximal indicates ±2 Kb from TSS and distal indicates sites that are further than that. **e**, Percentage of each TF's binding sites that are overlapped with those of p300 (common) or not (unique). **f**, Heatmaps showing occupancy signals of TFs around the center of all p300 peaks from both TSCs and ESCs. Overlap analysis of p300 binding sites separated three distinct classes of p300 sites (TSC-specific, common and ESC-specific) between TSCs and ESCs. ChIP-seq signals of each TF were mapped to the classes. **g**, Occupancy signals of CTCF around SEs.

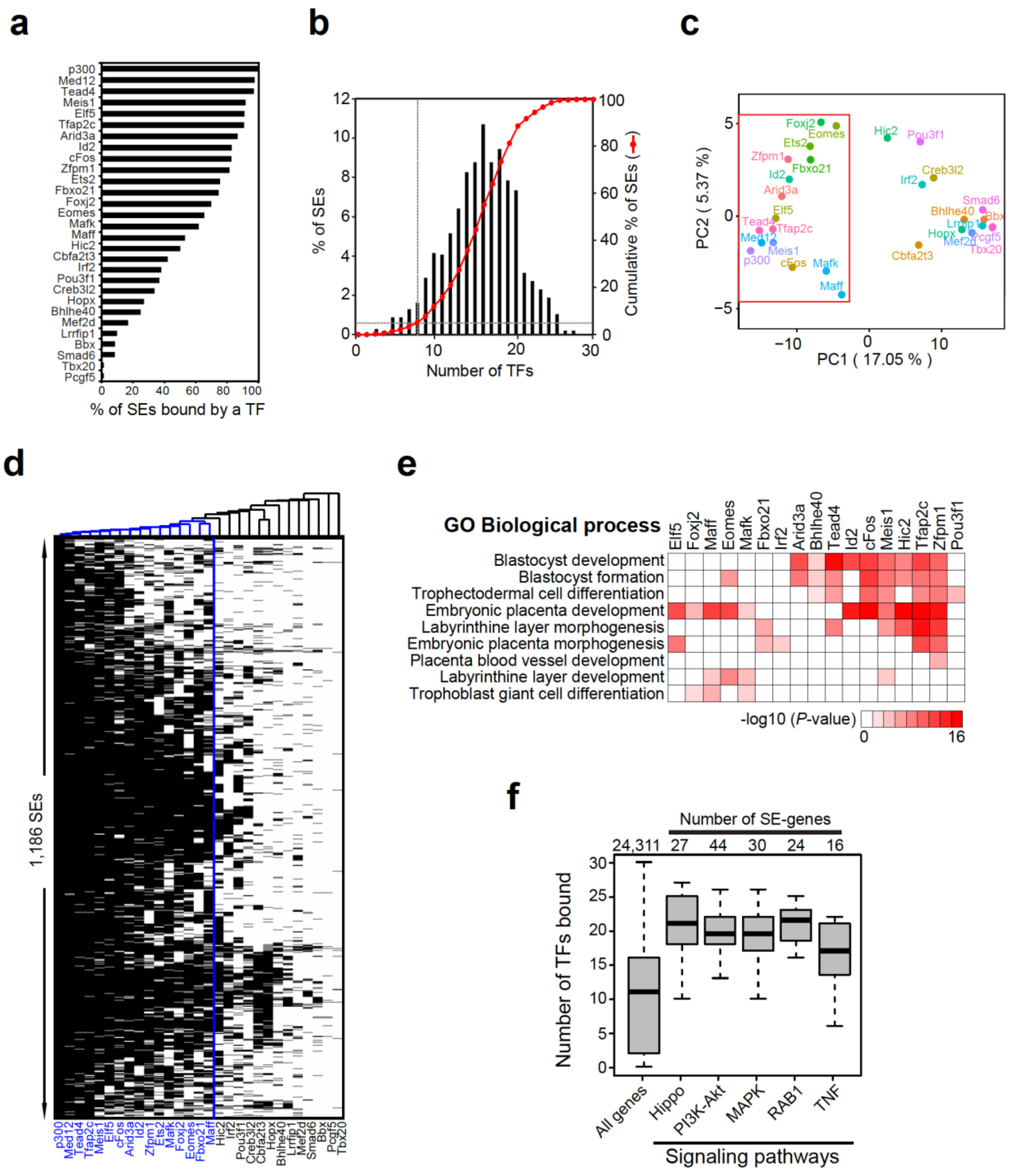

Supplementary Fig. 4. Functional roles of novel TSC-specific TFs in TSC. a, A bar graph showing percentage of SEs bound by a TF. **b**, A bar graph presenting percentage of SEs co-bound by a given number of TFs. A red line indicates cumulative percentage of SEs co-occupied by a given number of TFs. A cross point between vertical and horizontal dotted lines indicates a mark of 5% of cumulative SEs bound by less than 8 TFs. **c**, Principal component analysis (PCA) analysis of TFs' binding sites in SEs. A red box indicates 14 TFs that show the similar occupancy. **d**, Heatmap

presenting targets (black) and non-targets (white) of a TF for SEs. Blue indicates TFs that shows strong co-occupancy in SEs. **e**, A heatmap demonstrating enriched GO terms of biological process for the targets of TFs. **f**, A boxplot showing total number of TSC-specific TFs that occupy genes implicated in diverse signaling pathways. Numbers of genes that are not only involved in each signaling pathway but also bound by multiple TSC-specific TFs are shown on the top of the plot.

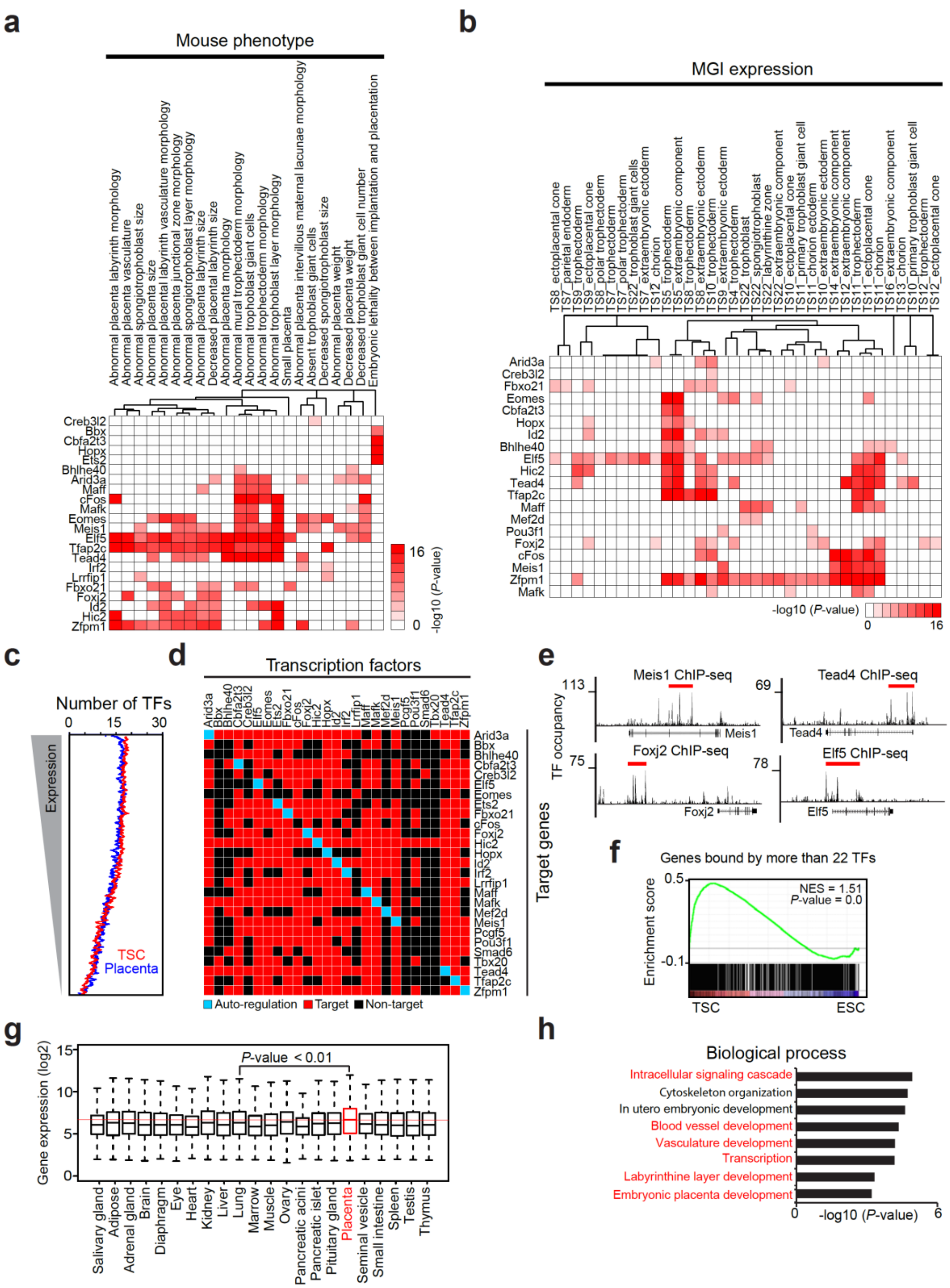

Supplementary Fig. 5. Regulatory modes of TSC-specific TFs in TSC. a, **b**, A heatmap demonstrating enriched GO terms of Mouse phenotype (**a**) and MGI expression (**b**) for the targets of TFs. **c**, A line graph presenting effects of number of TFs' binding on their target gene expression

in TSCs (red line) and Placenta (blue line). **d**, Auto-regulatory map of TSC-specific TFs. A TF that auto-regulates is labeled with sky blue. Targets and non-targets of a TF are labeled with red and black, respectively. **e**, ChIP-seq signal tracks of various TFs that auto-regulate. Red bar indicates a SE. **f**, GSEA of genes co-occupied with more than 22 TFs. NES indicates normalized enrichment score. **g**, A boxplot showing expression distribution of the genes that are co-occupied by more than 22 TFs in various mouse tissues. **h,** Enriched GO terms of biological process for the genes that are co-occupied by more than 22 TFs.

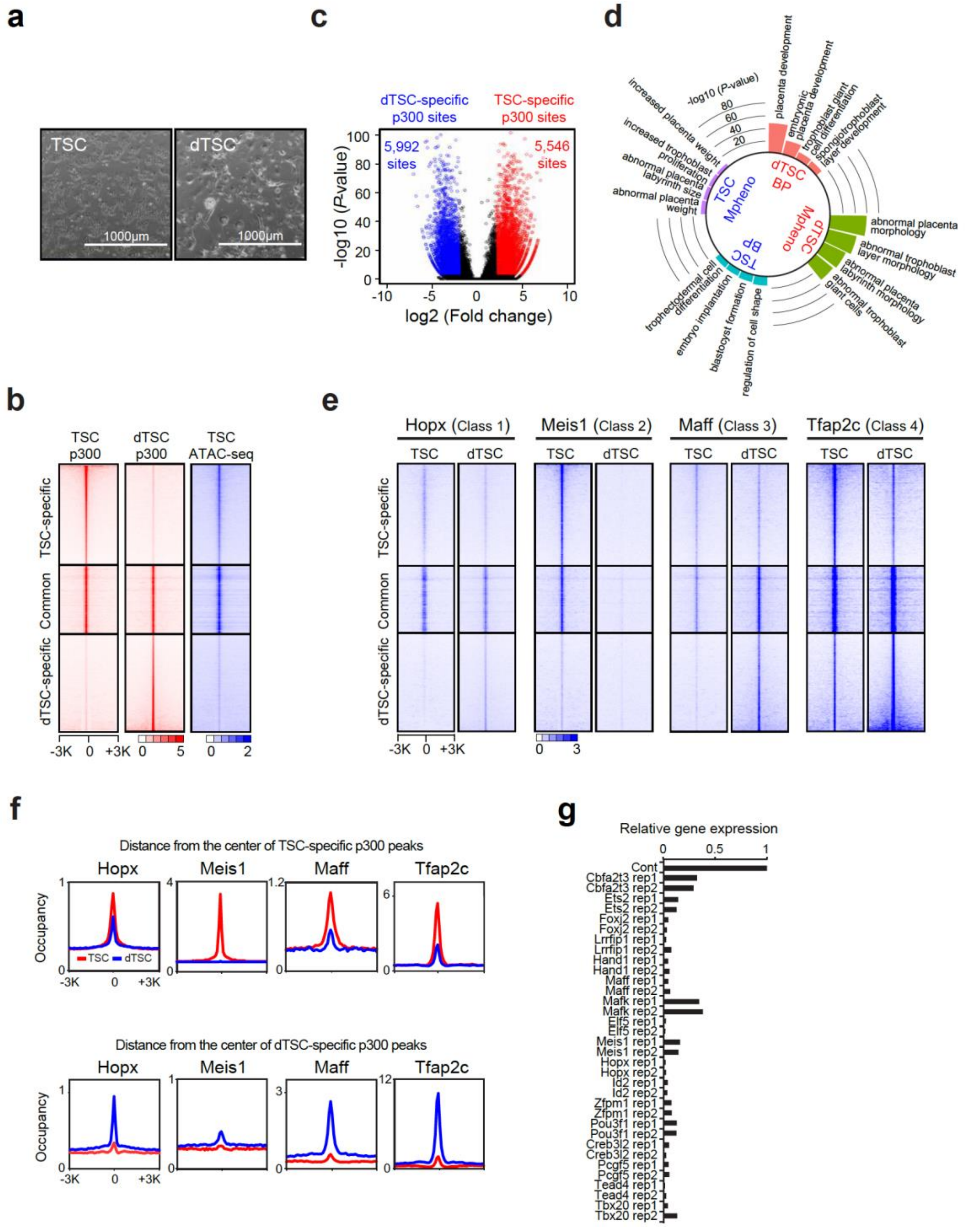

Supplementary Fig. 6. Context-dependent binding of TSC-specific TFs leads to distinct role of novel TSC-specific TFs. a, Bright field images of TSCs and differentiated trophoblast stem cells (dTSC) for 7 days. **b**, Heatmaps showing chromatin openness (right) and occupancy signals of p300 in TSC-specific, common and dTSC-specific p300 sites in TSCs (left) as well as dTSCs (center). **c**, A volcano plot

demonstrating statistically significant (fold change > 4 and FDR < 0.01) TSC-specific and dTSC-specific p300 sites. **d**, Enriched GO terms of biological process and mouse phenotype for the target genes of TSC-specific and dTSC-specific p300. **e**, Heatmaps showing occupancy signals of a representative TF among 4 classes in TSC-specific, common and dTSC-specific p300 sites in TSCs and dTSCs. **f**, Line graphs showing average occupancy score distribution of a TF near the center of TSC-specific (top) and dTSC-specific (bottom) p300 binding sites. Red and blue lines indicate ChIP-seq signal of TFs in TSC and dTSC, respectively. **g**. A bar graph presenting knockdown efficiency of tested TFs.

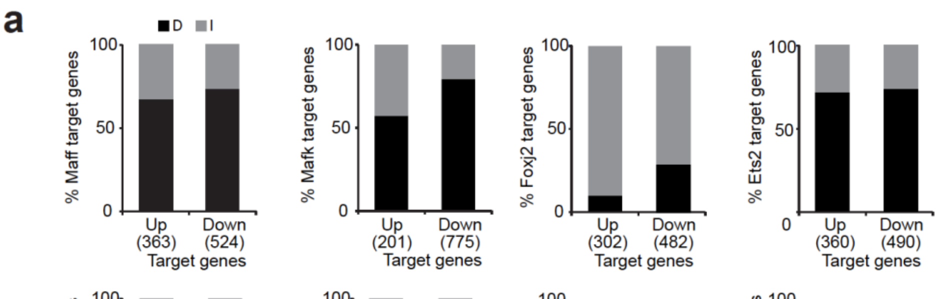

Supplementary Fig. 7. TSC-specific TFs directly regulates TE lineage differentiation. **a**, Numbers of up- or down-regulated direct and indirect targets of TSC-specific TFs. **b**, Enriched GO terms in differentially expressed genes (DEGs) that are directly regulated by TSC-specific TFs.

a

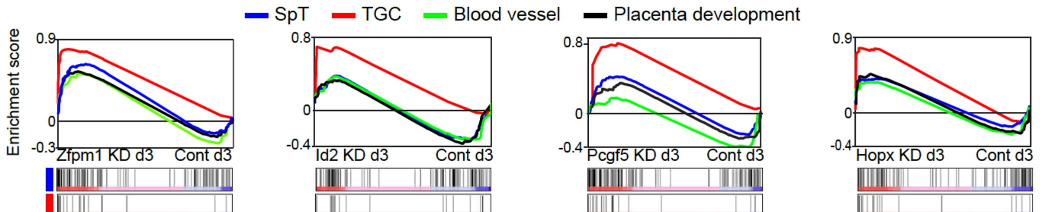

Supplementary Fig. 8. GSEA and IHC support class-dependent roles of TSC-specific TFs. a, **b**, Gene set enrichment analysis (GSEA) using global gene expression profiles obtained from KD of TFs with multiple gene sets representing specific placenta-related terms (**a**) and gene sets representing 28 placenta-specific single cells (**b**).

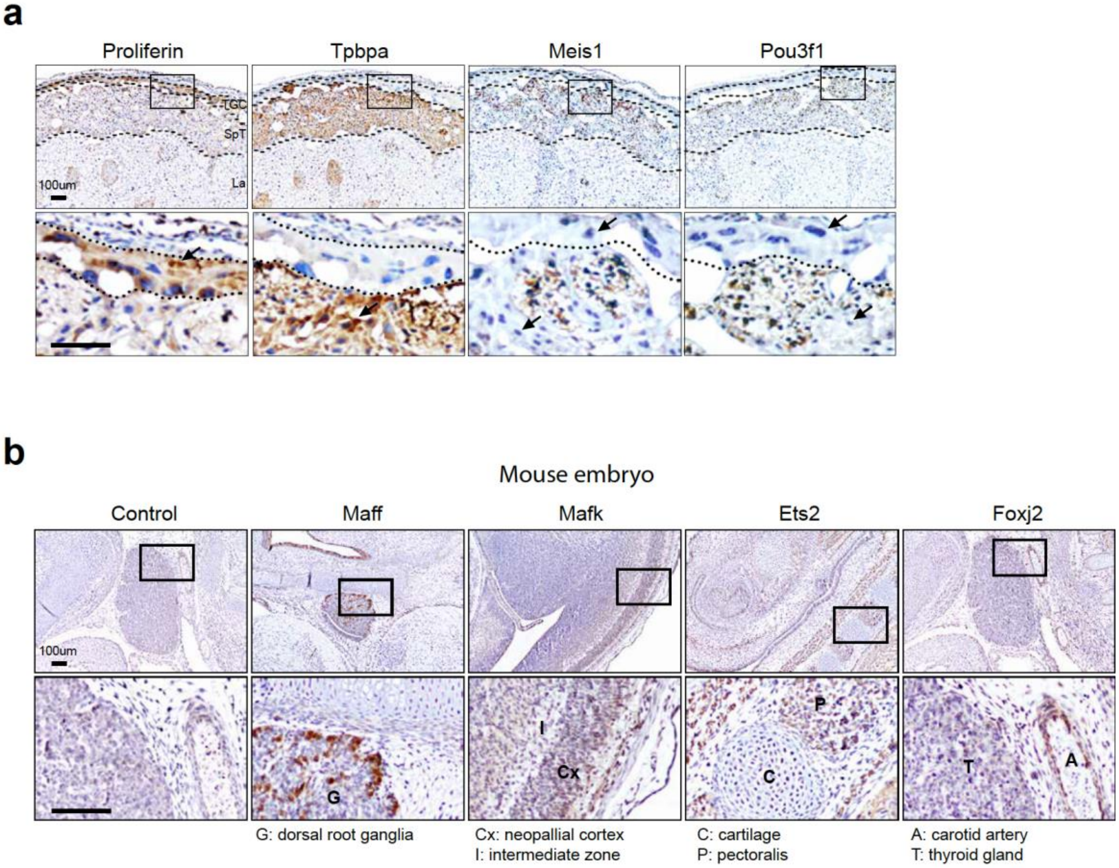

Supplementary Fig. 9. Immunohistochemistry of TGC markers and TSC-specific TFs. a, Immunohistochemistry of TGC marker (proliferin), SpT marker (Tpbpa), Pou3f1, and Meis1 in E15 mouse placenta. TGC, SpT, and La indicate trophoblast giant cell, spongiotrophoblast, and labyrinth, respectively. An arrow indicates a representative cell in trophoblast lineage and a scale bar depicts 100 µm. **b**, Immunohistochemistry of TSC-specific TFs in mouse embryo (E15). Each antibody recognizes a specific tissue of mouse embryo section. Ets2 and Foxj2 antibodies can recognize pectoralis and carotid artery, respectively. Maff antibody can specifically recognize dorsal root ganglia, while the Mafk antibody can detect the neopallial cortex.

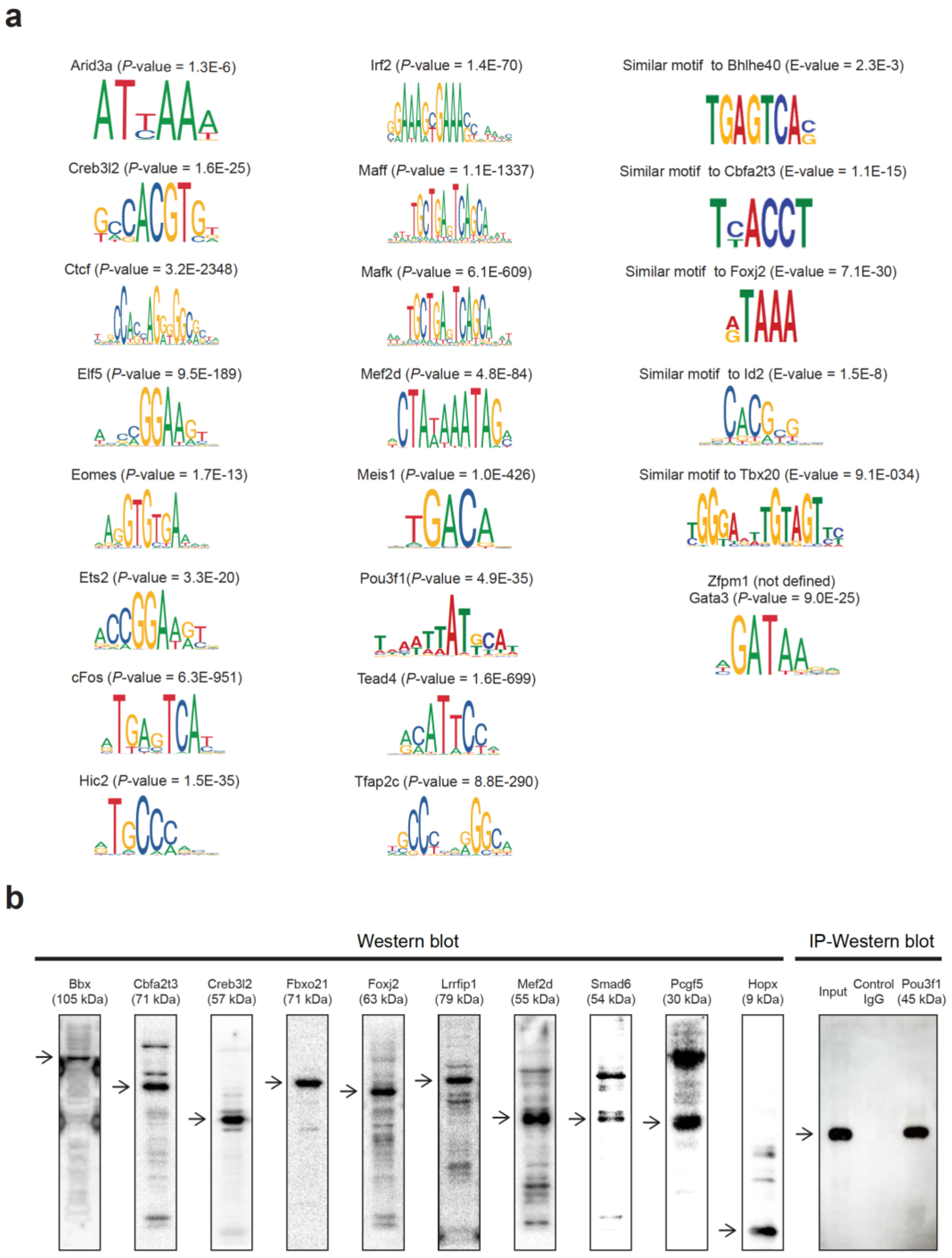

Supplementary Fig. 10. Antibody validations of TSC-specific TFs. a, Representative motifs enriched in the binding sites of TFs. **b**, Western blot or immunoprecipitation followed by Western blot (IP-Western) for several different antibodies. An arrow indicates the position of the protein of interest. Source data are provided as a Source Data file.
