# Supplementary material for: Super-enhancer-guided mapping of regulatory networks controlling mouse trophoblast stem cells
Source: Nat Commun. 2019 Oct 18;10:4749. doi: 10.1038/s41467-019-12720-6 (PMC6802173; doi:10.1038/s41467-019-12720-6)
Supplement: Supplementary file 3 — Description of Additional Supplementary Files [file 41467_2019_12720_MOESM3_ESM.pdf]

Description of Additional Supplementary Files

File Name: Supplementary Data 1
Description: p300 binding sites in TSCs

File Name: Supplementary Data 2
Description: p300 binding sites in ESCs

File Name: Supplementary Data 3
Description: Super enhancers in TSCs

File Name: Supplementary Data 4
Description: SE- and RE-associated genes in TSCs

File Name: Supplementary Data 5
Description: Transcriptional regulators that are associated with SEs in TSCs

File Name: Supplementary Data 6
Description: Known and unknown TSC-specific TFs

File Name: Supplementary Data 7
Description: Targets of each TF in TSCs

File Name: Supplementary Data 8
Description: SE-associated genes showing abnormal expressions in human PE

File Name: Supplementary Data 9
Description: TSC-specific genes bound by more than 22 TFs

File Name: Supplementary Data 10
Description: Validation of antibodies

File Name: Supplementary Data 11
Description: Sequences of primers, shRNAs, antibodies, and data deposited or used for analyses
